# A prospective, internal validation of an emergency patient triage tool for use in a low resource setting

**DOI:** 10.1016/j.afjem.2022.05.003

**Published:** 2022-06-24

**Authors:** Brian Kikomeko, George Mutiibwa, Pauline Nabatanzi, Alfred Lumala, John Kellett, Kitovu Hospital Study Group

**Affiliations:** a Emergency and out-patient department, Kitovu Hospital, Masaka, Uganda; b Emergency and out-patient department, Kitovu Hospital, Masaka, Uganda; c Medical Ward Sister, Kitovu Hospital, Masaka, Uganda; d Kitovu Hospital, Masaka, Uganda; e Department of Emergency Medicine, Hospital of South West Jutland, Esbjerg, Denmark

**Keywords:** Triage low resource setting predictive scores, Emergency department

## Abstract

**Aim:**

Assess the performance of a simple triage disposition score based on mental status, mobility and either oxygen saturation or respiratory rate by three principal metrics: 24 h mortality, the need for hospital admission and the urgency ranking of patient presentations.

**Method:**

Prospective observational non-interventional study of consecutive patients presenting to the emergency and outpatient departments of a low-resource sub-Saharan hospital

**Results:**

Out of 14,585 consecutive patients arriving to hospital 1,804 (12.4%) were admitted and 39 died (0.3%) within 24 hours. No patients with normal mental status or a stable independent gait died within 24 h, and 95% of those who did had an oxygen saturation <94%. The c statistic of the score for death within 24 hours was >0.95 and not significantly changed if respiratory rate replaced oxygen saturation as a score component, or mental status was assessed subjectively or objectively. However, an objective measure of mental status significantly reduced the c statistic for hospital admission from 0.970 SE 0.003 to 0.956 SE 0.004, *p* 0.002. The score attributed a higher acuity rating than the South African Triage System urgency ranking of presentations to 11.1% of patients and a lower acuity rating to 1.3%. However, 53% of the patients given a higher acuity rating were subsequently admitted to hospital and 6.1% of them died.

**Conclusion:**

The score identified patients who subsequently required hospital admission and who were likely to die within 24 hours.

## African relevance

• In a low-resource hospital in Uganda a simple triage disposition score identified patients likely to die within 24 hours
• In a low-resource hospital in Uganda a simple triage disposition score identified patients requiring admission to hospital
• In a low-resource hospital in Uganda a simple triage disposition score correlated well with the South African Triage Scale urgency rankings
• In a low-resource hospital in Uganda a simple triage disposition score was easy to perform, and required little training or equipment

## Introduction

The purpose of triage is to identify those patients who need immediate attention. The Australasian Triage Scale, Canadian Triage and Acuity Scale, Emergency Severity Index, Manchester Triage Scale, and South African Triage Scale (SATS) are the most widely adopted. They were all developed by consensus opinion, and they all rely on some level of subjective judgment by trained healthcare workers [1]. Additional triage systems for low-resource settings have also been proposed [2]. The World Health Organization, International Committee of the Red Cross, and Médecins Sans Frontières have developed the Integrated Interagency Triage Tool (IITT) for use in resource-limited emergency centres. In Papua New Guinea [3] IITT detected time-critical diagnoses and identified patients likely to die or require admission to hospital. However, IITT, has 20 very urgent (RED) criteria, with 8 additional ones if the patient is pregnant. Moreover, some of its criteria, such as capillary refill time, heart rate, hypothermia, blood pressure, ECG changes and the assessment of mental status are all likely to take time, equipment, and expertise. IITT, therefore, may be difficult to use in many low-resource settings [4].

The SATS that has been widely implemented and evaluated in South Africa, in several low-or middle-income countries, and in a wide range of settings [5]. Although it was designed for nursing assistants [6], some nursing staff in a Ugandan hospital found it difficult to use [7]. Since some conditions, such as severe pain, require immediate attention even if they are not life-threatening, SATS assigns arbitrary rankings of urgency for specific patient presentations [8]. If a patient is not ranked urgent, very urgent, or emergent by their clinical presentation, the patient should be further evaluated by measuring a full set of vital signs, which is time consuming [9] and requires equipment used by trained conscientious staff [10], followed by accurate calculation of the Triage Early Warning Score (TEWS) [11].

As part of an ongoing quality improvement project, the Kitovu Hospital Study Group used inpatient data to derive and validate the Kitovu Hospital Disposition Score (KHDS); the score awards one point for altered mental status, one point for impaired mobility, and one point for either a low oxygen saturation or an increased respiratory rate. KHDS was derived and validated using data collected *after* patients were admitted to hospital and not on all patients who presented to the hospital at the time of their arrival. Moreover, the only metric used to assess its performance was mortality prediction, which may not be the best surrogate metric for a triage [12]. Alternative metrics include the need for hospital admission, the prompt relief of suffering, the recognition of conditions requiring time-critical treatments, and the resources a triage system consumes [1,13,14].

The aim of this study was to demonstrate the performance of KHDS in practice using data collected on non-obstetric patients at their time of arrival to hospital. A particular concern was that KHDS might miss some patients with a high SATS urgency presentation. KHDS performance was assessed by three metrics: 24 h mortality, the need for hospital admission and the urgency ranking of patient presentations.

## Methods

Demonstration of the KHDS's performance in a low-resource setting by three metrics: 24-hour mortality, the need for hospital admission and the urgency ranking of patient presentations.

This prospective cross-sectional observational non-interventional study was performed in the emergency and outpatient departments of Kitovu Hospital, which has 248 beds (50 medical and 35 surgical) and is located near Masaka, Uganda. The emergency centre (EC) and outpatient departments (OPD), which care for all patients attending the hospital except those attending the obstetric department, are located beside each other, sharing a common entrance and clinical staff who move between them as needed. Most emergency medical care is provided by recently qualified doctors (within 3 years of graduation) assisted by clinical officers (non-physician clinicians) [15].

The emergency centre is open 24 h a day and the outpatient department from 9am to 5pm. After arrival, patients are directed to either the emergency centre or the outpatient clinic by an informal process, depending on patient wishes, staff availability and their judgement, crowding, time of day etc. During the day, the combined departments are staffed by at least two clinical officers and a doctor; at night, one doctor is first on-call and supported by two others who are second and third on-call. Twice a week, there are outpatient clinics attended by visiting consultant specialists.

The study was part of an ongoing audit process, and its size and duration were arbitrarily determined by the resources available. Participants were all non-pregnant patients aged 12 years or older who consecutively attended the combined OPD/EC from 23^rd^ November 2020 to 31^st^ October 2021. There were no other exclusion criteria. During the day, a dedicated researcher entered patients’ age, sex, date, time of arrival, respiratory rate and/or oxygen saturation, mobility, mental status, and SATS urgency ranking into an Excel database (Version 2102, Microsoft Corp., Redmond, WA). This was a manual system: the exact complaint associated with each urgency ranking was entered along with additional information considered to be important in free text. At night, this information was recorded by the nurse on duty on paper and transcribed into the database the following morning. This information was then reviewed to ensure that the clinical description of each SATS ranking was correct. The subsequent immediate disposition of each patient was also recorded (i.e., admitted, discharged, or died while in the emergency centre), and hospital records were then reviewed to identify patients who died while in hospital. The clinical staff caring for the patient had no access or knowledge of the data collected or the study purpose and all their management decisions were made independently of it.

Throughout the study the default KHDS awarded one point for altered mental status, one point for impaired mobility and one point for an oxygen saturation <94%. Assessments of respiratory rate, alternative evaluations of mental status and SATS urgency rankings were introduced as the study progressed (Table 1).

**Table 1:** The starting dates and number scores calculated on presenting patients of different variations of the Kitovu Hospital Disposition Score (KHDS) and the South African Triage Scale discriminators compared, according to age, sex, admission rates and 24-hour mortality rates of presenting.

	KHDS usingOxygen saturation	KHDSm usingmonths backwards	South African Triage Scale discriminators	KHDSr using respiratory rate
***Starting date***	*23 November 2020*	*03 February 2021*	*01 May 2021*	*23 June 2021*
***Number calculated***	*14,585*	*11,481*	*7,500*	*4,368*
**Age (years)**	44.0 SD 19.9	43.7 SD 19.8	44.0 SD 19.9	44.4 SD 19.5
*Median*	*40*	*40*	*40*	*41*
*IQR*	*28-59*	*28-58*	*28-58*	*29-58*
**Male sex**	5,711 *(39.2%)*	4,481 *(39.0%)*	2,902 *(38.7%)*	1,653 *(37.8%)*
**Admitted**	1,804 *(12.4%)*	1,376 *(12.0%)*	979 *(13.1%)*	635 *(14.5%)*
**Died within 24 hours**	39 *(0.3%)*	26 *(0.2%)*	19 *(0.3%)*	10 (*90.2%)*

Impaired mobility on presentation was defined as lack of a stable independent gait. Therefore, any patients unsteady on their feet, needed a walking stick or other aid to steady themselves, help to walk or were bedridden were considered to have an impaired mobility. Oxygen saturation and heart rate were measured by the Acc U Rate CMS 500D finger oximeter (CMS Mobility, Stafford, USA), which required to 30 to 60 seconds to obtain a stable reading.

Assessment of mental status was subjective; patients were considered to have normal mental status if during conversation they were alert, attentive, calm, and coherent. From February 3^rd^, 2021, an additional version of the disposition score (KHDSm) was also used, which defined normal mental status as the ability to count the months of the years backwards from December to July [16].

From May 1^st^, 2021, presentations were ranked by SATS as emergent, very urgent, urgent, or non-urgent [8]. Reduced level of consciousness was re-defined as coma (i.e., responsive to pain or unresponsive). Other presentations could not be recorded as they were beyond the diagnostic expertise available (e.g., post-ictal, compound fracture, dislocations, etc) or were not observed (e.g., stabbings, eye injuries etc). If a patient presented with more than one SATS urgency ranked presentation their urgency ranking was determined by the presentation with the highest ranking.

From June 23^rd^, 2021, KHDS was also calculated using a respiratory rate >23 breaths per minute (KHDSr). Respiratory rates were measured by the *RRate* app [17], which is available free from public app stores [18,19]. The application's screen displays a large button that is tapped every time the patient inspires, and its algorithm calculates the respiratory rate based on the interval time between taps.

Preliminary data on the discrimination of KHDS for hospital admission based on data collected between November 2020 and March 2021 has already been published [20]. Numeric variables were compared using Student's t test and categorical variables were compared using chi squared analysis with Yates’ continuity correction, when applicable; calculations were performed using Epi Info, version 6.0 (Centres for Disease Control and Prevention, Atlanta, USA). The p value for statistical significance was 0.05. The C statistic was used to assess the discrimination of the score for hospital admission according to the method of Hanley and McNeil [21].

Ethical approval of the study was obtained from the scientific committee at Kitovu Hospital. The study conforms to the principles outlined in the Declaration of Helsinki [22]. The study is reported in accordance with the STROBE statement [23].

## Results

From November 23^rd^, 2020, to October 31^st^, 2021, 14,585 patients (43 patients per day, mean age 44.0 SD 19.9 years) had their mental status, gait and oxygen saturation assessed on hospital arrival, from which the default KHDS based on oxygen saturation was determined; 5,711 (39.2%) were men; 1490 (10.2%) scored 1 point, 1805 scored 2 points (12.4%) and 653 scored 3 points (4.5%). Of the 1,804 (12.4%) patients who were admitted to hospital, only 96 (5.3%) had a KHDS less than two points.

**Fig. 1:**
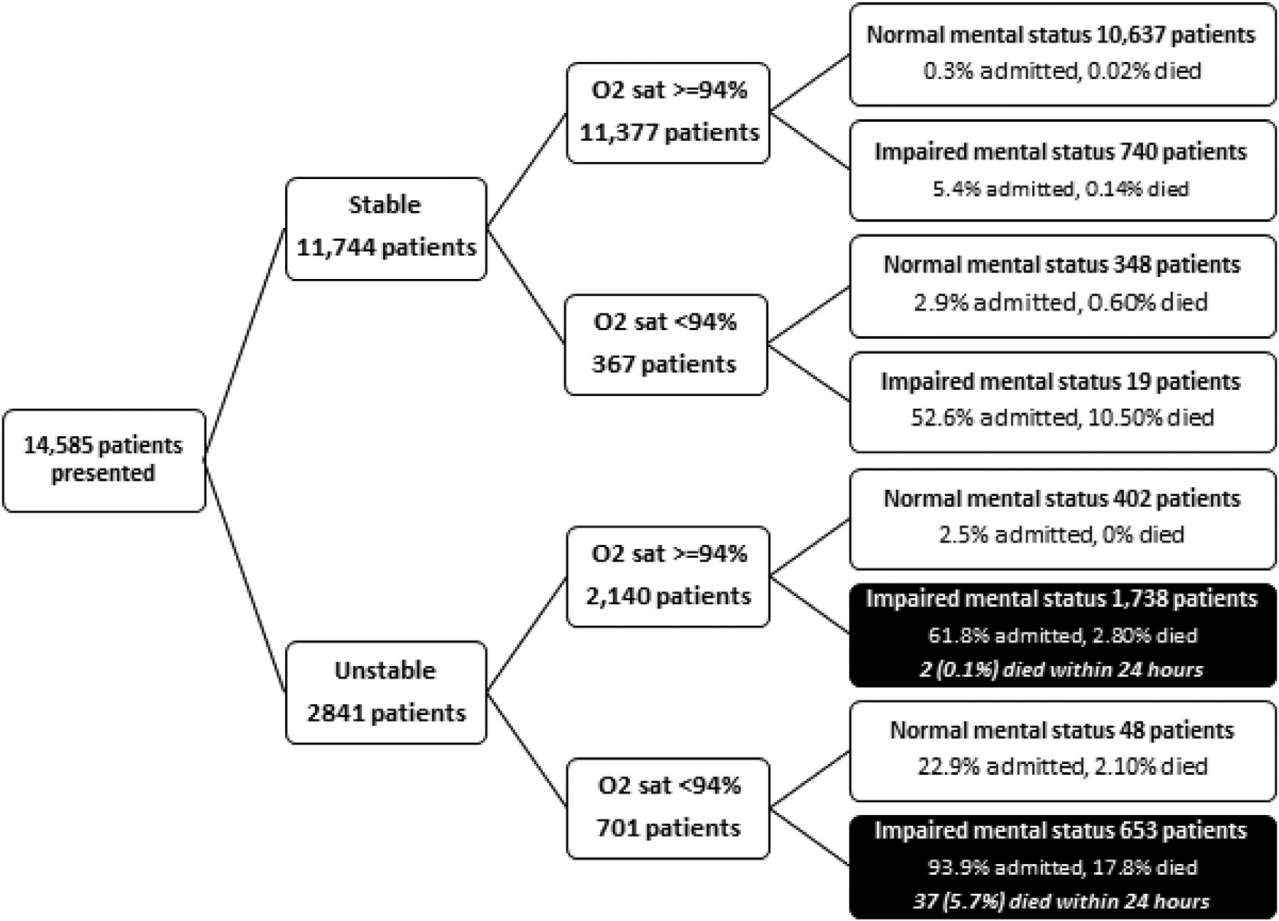
Hospital admission and in-hospital mortality according to gait, oxygen saturation and mental status at presentation. Patients who were alert, attentive, calm and coherent had a normal mental status, otherwise they had impaired mental status.

All patients who died were counted as hospital admissions. One hundred and seventy-three patients died in hospital (1.1% of all presentations and 9.6% of all admissions). Thirty-nine patients died within 24 hours of hospital arrival (0.3% of all presentations and 2.2% of all admissions); 37 had a KHDS of three points, and two had two points. After 24 hours 134 more patients who were admitted to hospital died, 93 of them (69.4%) within 5 days. None of the 11,744 (80.5%) patients with a stable gait or the 11,435 (78.4%) patients who were alert, attentive, calm, and coherent died within 24 hours (Fig. 1). The c statistic of KHDS for death within 24 hours was 0.975 SE 0.018, and 0.965 SE 0.003 for hospital admission. There was no significant difference in the c statistics between men and women for mortality (0.964 SE 0.028 versus 0.982 SE 0.022, p 0.34) or hospital admission (0.964 SE 0.004 versus 0.967 SE 0.004, p 0.29).

Mental status, mobility and oxygen saturation were all statistically associated (i.e., *p* <0.0001) with death within 24 h, hospital admission and a SATS urgency ranking. There was no statistical difference in the c statistics for death within 24 h of versions of KHDS that used oxygen saturation, respiratory rate, or the months backwards test. The SATS ranking for urgency also had the same discrimination for death within 24 h. However, the discrimination for admission to hospital was significantly lower for the SATS rankings, and for assessment of mental status by the months backwards test (Table 2).

**Table 2:** Comparison of the discrimination of the South African Triage Scale (SATS) presentation urgency rankings and different configurations of the Kitovu Hospital Disposition Score (KHDS) using oxygen saturation (KHDS), respiratory rate (KHDSr) or the months backwards test (KHDSm) to assess mental status.

	*n*	C statistic	SE	*compared to:*	C statistic	SE	p
**Death within 24 hours**							
KHDS - using oxygen saturation	*14,585*	0.975	0.018				
KHDSr - using respiratory rate	*4,368*	0.954	0.046	*KHDS - using oxygen saturation*	0.971	0.038	0.39
KHDSm - using months backwards test*	*11,481*	0.982	0.018	*KHDS - using oxygen saturation*	0.978	0.020	0.44
SATS presentation urgency rankings	*7,500*	0.900	0.048	*KHDS - using oxygen saturation*	0.977	0.024	0.07
**Hospital admission**							
KHDS - using oxygen saturation	*14,585*	0.965	0.003				
KHDSr - using respiratory rate	*4,368*	0.970	0.005	*KHDS - using oxygen saturation*	0.969	0.005	0.44
KHDSm - using months backwards test*	*11,481*	0.956	0.004	*KHDS - using oxygen saturation*	0.970	0.003	0.002
SATS presentation urgency rankings	*7,500*	0.792	0.009	*KHDS - using oxygen saturation*	0.970	0.004	<.0001

*KHDSm used oxygen saturation; n = patient number; SE = standard error

**Fig. 2:**
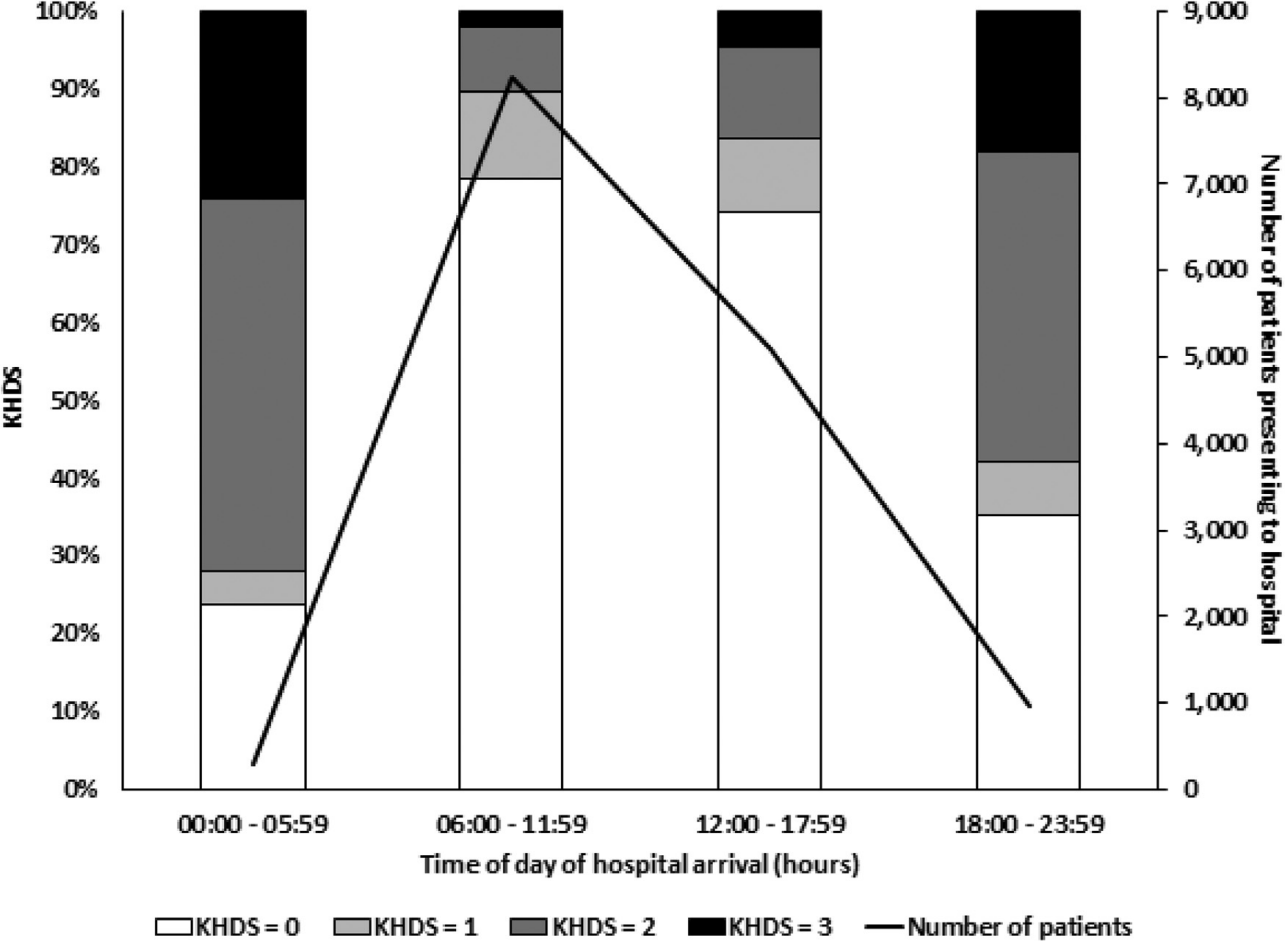
Kitovu Hospital Disposition Score by time of patient presentation. KHDS = Kitovu Hospital Disposition Score

KHDS on hospital arrival was associated with the time of day (Fig. 2). Twenty (0.2%) of the 13,316 patients presenting to hospital between 6 am and 6 pm died within 24 h, compared with 12 (4.4%) of the 297 patients who presented between midnight and 6 am.

KHDS and 24 h mortality both increased with age; the number of patients presenting with a score of three increased exponentially from 2% at age 20 to 23% by 90 years of age, and between these ages 24 h mortality also increased 10-fold from 0.13% to 1.30%.

Of the 7,500 presenting patients ranked for urgency by SATS, none with a disposition score of zero were rated as urgent, very urgent, or emergent presentations, and only 7 of 735 (1%) patients with a disposition score of one had an urgent or very urgent presentation (Table 3); none of these patients died within 24 hours. The only SATS urgency presentations significantly associated with 24-hour mortality were hypoglycaemia, pre-arrest, breathlessness, coma, diabetes with or without ketoacidosis. Out of 6,926 patients with non-urgent SATS rankings 768 (11.1%) had a KHDS of >= 3 points; three of these patients (0.4%) died within 24 hours, 407 (53.0%) were admitted to hospital where 22 (5.4%) of them subsequently died more than 24 hours after admission. Only 7 (1.3%) of 551 patients ranked urgent or very urgent by SATS had KHDS of 1 point (Table 3); all of these patients were admitted and none of them died.

**Table 3:** South African Triage System (SATS) presentation urgency rankings observed according to the Kitovu Hospital Disposition Score (KHDS). * Reduced level of consciousness was re-defined as coma (i.e., responsive only to pain or unresponsive). Other presentations were not recorded as they or required expertise that was not available (e.g., post-ictal, compound fracture, dislocations, etc). Other were not observed (i.e., facial burns, inhalation, stab wounds, eye injuries, pregnancy related complaints).

	Kitovu Hospital Disposition Score points:				
South African Triage Scale Presentation ranking	ZERO	ONE	TWO	THREE	*Total
***Emergent***					
Hypoglycaemia	0	0	11	2	13
Apnoea/Pre-arrest	0	0	0	8	8
Fitting	0	0	4	0	4
Total*	***0***	***0***	***15***	***8***	***23***

***Very urgent***					
Severe breathlessness	0	0	10	139	149
Head injury - open wound and/or suspected skull fracture	0	1	61	7	69
High energy transfer injury	0	1	56	6	63
Severe pain	0	1	38	11	50
Suspected stroke	0	0	33	12	45
Haemoptysis or Uncontrolled bleeding	0	0	26	7	33
Coma (responsive only to pain or unresponsive) *	0	0	15	14	29
Diabetic keto-acidosis	0	1	17	9	27
Burns	0	1	3	0	4
Poisoning	0	0	4	0	4
Chest pain	0	0	1	3	3
Total*	***0***	***5***	***206***	***184***	***395***

***Urgent***					
Abdominal pain	0	2	106	37	145
Diabetes without keto-acidosis	0	0	33	22	55
Moderate pain	0	0	44	6	50
Suspected fracture	0	0	24	0	24
Controlled bleeding	0	0	6	0	6
Vomiting	0	0	2	0	2
Total*	***0***	***2***	***127***	***27***	***156***

***Non-urgent***					
***Total***	***5,430***	***728***	***689***	***79***	***6926***

⁎ Totals are for the KHDS, many patients had more than one SATS urgency presentation

## Discussion

KHDS identified patients who were likely to die within 24 h, to require admission to hospital, and the likely SATS urgency ranking of their clinical presentation. All the modifications of the KHDS tested identified patients who were likely to die within 24 h and, therefore, needed immediate attention. None of the patients with normal mental status or a stable independent gait died within 24 h; 95% of those who did die within 24 h had an oxygen saturation <94%.

This unfunded study was based on information that could be easily obtained from patients when they first presented to hospital. Therefore, the amount of information collected, and its detail, had to be limited. The study was confined to patients aged 12 years or older, was performed in a single centre and did not include obstetric patients, and some urgency presentations could not be recorded as they were beyond the diagnostic expertise available. Some SATS urgency ranking presentations were not observed during the study. Therefore, some special populations in need of time critical treatment, such as penetrating injuries, were not included and might possibly be missed by KHDS. We did not record or consider the number of patients who attended repeatedly and were unable to follow-up patients after discharge from the hospital. It is unlikely knowledge of our publication of the discrimination of KHDS for hospital admission based on data collected prior to 30 March 2021 [20] influenced the hospital's admission practice as there was no change in admission rates before and after March 30^th^, 2021 (i.e., 12.1% versus 12.6%).

Demonstrating that any triage system is beneficial is problematic, and most validation studies have used either the utilisation of resources or a patient outcome, such as mortality or hospital admission as a proxy metric [1,13,14,24]. The ultimate benefit of triage must be improved patient outcomes, which may include reduced mortality, more rapid relief of pain and discomfort, and reduced morbidity. Even if these patient-specific benefits cannot be demonstrated, a triage system may allow the same quality of care to be delivered at a lower cost by fewer staff who require less training and skill. These benefits must be balanced against the costs and resources triage consumes.

Validating a triage system by showing it reduces mortality is difficult because, apart from major disasters or mass casualty incidents, the chance of imminent death for any patient is low and many are unpreventable; the 0.3% 24 h mortality observed in this study is comparable to most reports from emergency centres and acute hospital settings in the literature [25,26]. The discrimination of KHDS for death within 24 h is comparable to the National Early Warning Score (NEWS), which is currently the most widely used and best validated method of identifying patients at risk of imminent death [26].

In a low resource or disaster setting anyone should be able to perform triage quickly and easily without training or expensive, complicated equipment. KHDS resembles the Simple Triage and Rapid Treatment (START) system used in major disasters or mass casualty incidents, which first assesses ability to walk, then determines if there is spontaneous breathing, followed by measurements of respiratory rate, radial pulse, capillary refill and if the patient can obey commands. However, against expert opinion START attributed a lower acuity rating to 10% of patients and a higher acuity to 14% [27]. In contrast, compared with SATS urgency rankings KHDS attributed a lower acuity rating to only a tiny number of patients.

KHDS was easy to use, took little time, skill, or training, and did not significantly increase the workload of OPD/EC staff; only 1.9 patients per day present with three points and 5.3 per day with two points. Subjective assessment of mental status discriminated the subsequent need for hospital admission better than using the more objective “months backwards” test [16] and was also superior to the SATS presentation rankings.

A major challenge for any triage system is identifying patients with time-critical illness who do not seem that sick. Arguably the higher acuity ratings attributed by KHDS reflects an inappropriately low rating by the SATS rankings, as 53% of those with a non-urgent ranking and a high KHDS were admitted and 6.1% died in hospital). Some SATS presentations, such as diabetic keto-acidosis and hypoglycaemia, require clinical knowledge and/or waiting for the results of an investigation. Nevertheless, defining a patient's bedside SATS presentation should help direct immediate treatment, such as protection of the airway, positioning of the patient, circulatory support, control of bleeding, relief of pain etc.

Unlike patients with a low NEWS, who remain clinically stable with a low risk of death for several days [26], patients with a low KHDS can rapidly deteriorate; 96 out of the 12,031 (0.8%) patients with a KHDS <2 points were assessed to need hospital admission and three (3.1%) of these patients died within 4 days. Therefore, KHDS should not replace measurement of a complete set of vital signs but may help prioritize when it and a full clinical assessment should occur. KHDS, SATS rankings and TEWS should be used to complement each other, depending on the time, skills, and resources available.

## Conclusion

KHDS identified patients who were likely to die within 24 hours, required admission to hospital, and the urgency ranking of their clinical presentation. KHDS has many of the characteristics of an ideal triage process for a low-resource setting, as it is easy to perform and uses equipment that is simple, cheap, available, and robust.

## Dissemination of results

Results from this study was shared with doctors and nurses working on our own hospital, and involving them in our findings, and encouraging them to make constructive criticisms and suggestions on how our findings should be implemented.

## Authors’ contributions

Authors contributed as follow to the conception or design of the work; the acquisition, analysis, or interpretation of data for the work; and drafting the work or revising it critically for important intellectual content: BK contributed 40%; JK 30%; GM 20%; and PN and AL contributed 5% each. All authors approved the version to be published and agreed to be accountable for all aspects of the work.

## Declaration of Competing Interest

John Kellett is a founder and major shareholder of Tapa Healthcare DAC, a start-up medical software company. The other authors have no conflict of interest.
